# Enhanced surface functionalization of 2D molybdenum/tin chalcogenide nanostructures for effective SERS detection of *Escherichia coli*†

**DOI:** 10.1039/d4ra05315j

**Published:** 2024-11-04

**Authors:** Zainab Ishfaq, Layla A. Almutairi, M. Yasir Ali, Salhah Hamed Alrefaee, Mohamed Abdelsabour Fahmy, Elsammani Ali Shokralla, Lamiaa G. Alharbe, Adnan Ali, Arslan Ashfaq, A. R. Abd-Elwahed

**Affiliations:** a Department of Physics, Government College University Faisalabad 38000 Pakistan adnnan_1982@yahoo.com arslan.ashfaq201@gmail.com; b Department of Biology, College of Science Princess Nourah bint Abdulrahman University P. O. Box 84428 Riyadh 11671 Saudi Arabia; c Department of Chemistry, Faculty of Science, Taibah University Yanbu 30799 Saudi Arabia; d Department of Mathematics, Adham University College, Umm Al-Qura University Adham 28653 Makkah Saudi Arabia; e Department of Basic Sciences, Faculty of Computers and Informatics, Suez Canal University New Campus 41522 Ismailia Egypt; f Department of Physics, Faculty of Science, Al-Baha University Alaqiq 65779-7738 Saudi Arabia; g Department of Physics, Aljamoum University College, Umm Al-Qura University Makkah Saudi Arabia; h Department of Physics, College of Science, Qassim University Buraydah 51452 Saudi Arabia

## Abstract

Surface Enhanced Raman Spectroscopy (SERS) is a highly sensitive analytical technique used for fingerprint recognition of molecular samples. The SERS effect, which enhances Raman scattering signals, has been the subject of extensive research over the past few decades. More recently, the commercialization of portable Raman spectrometers has brought SERS closer to real-world applications. The aim of the study was to enhance their performance, properties, and biocompatibility for potential use as SERS substrates. The synthesis and characterization of MoS_2_ and SnS_2_ nanoparticles are described, along with the functionalization process using l-cysteine. The detection and identification of *Escherichia coli* (*E. coli*) bacteria using MoS_2_ and SnS_2_ as SERS substrates are also investigated. The results demonstrate the successful functionalization and characterization of the nanostructures, indicating their potential as SERS substrates. The abstract highlights the importance of developing cost-effective and environmentally friendly disposable analysis chips with high accuracy and specificity for practical SERS applications.

## Abstract 2

This study enhances MoS_2_/SnS_2_ nanoparticles for SERS, functionalizing them with l-cysteine. Successful detection of *E. coli* demonstrates their potential as cost-effective SERS substrates for real-world applications.

## Graphical abstract

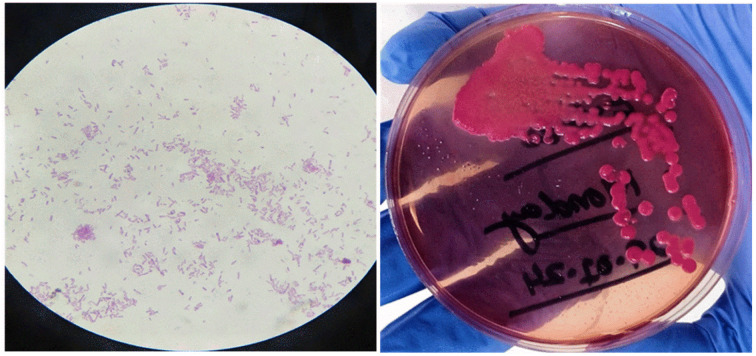

## Introduction

In recent times, Surface-Enhanced Raman Scattering (SERS) has gained significant recognition as an influential analytical method. SERS boasts an exceptional level of sensitivity, coupled with its ability to identify unique molecular fingerprints, thus facilitating the detection of analytes at extremely low concentrations.^1^ The phenomenon of surface enhancement is primarily elucidated by two main mechanisms: the localized surface plasmon resonance (LSPR) and the chemical or charge-transfer processes. These mechanisms provide a comprehensive understanding of the enhancement effect observed in SERS.^2^ Extensive research spanning several decades has focused on investigating the SERS effect.^3^ Notably, recent advancements in technology have brought about the commercialization of portable Raman spectrometers, thereby bringing SERS closer to practical applications in various fields. These applications include but are not limited to food safety and quality control, medical diagnostics, environmental monitoring and homeland security.^4,5^

In order to expand the practical utilization of SERS in real-life scenarios, it is imperative to develop cost-effective, environmentally friendly disposable analysis chips that offer both high accuracy and specificity.^6,7^ Additionally, it is crucial to mitigate the risks associated with cross-contamination and false positives. These factors play a crucial role in ensuring the reliability and effectiveness of SERS in various applications. Numerous techniques have been documented in the literature for fabricating structured SERS-active substrates, including dip coating, spin-coating, electrochemical synthesis, chemical vapor deposition, soft lithography, etching and electron beam lithography.^8–10^ However, it is important to note that these methods possess certain limitations with regards to either throughput volume or cost implications.^11,12^ In addition, achieving consistent signal intensity across different regions poses a significant challenge when it comes to the mass production of SERS-active nanostructures.^13,14^

The remarkable physical and chemical properties exhibited by 2D materials have sparked growing interest among researchers and scientists. 2D nanomaterials offer distinctive advantages, including facile synthesis, significant specific surface areas, remarkable mechanical properties, excellent optical properties, and favorable biocompatibility.^15,16^ These advantages contribute to their practical application in enhancing SERS and provide a viable solution to overcome challenges associated with metal substrates, such as high costs, catalytic effects, strong metal–adsorbate interactions, and photobleaching.^17,18^ As a result, numerous 2D materials have been extensively explored as potential SERS substrates.

Molybdenum disulfide (MoS_2_) has garnered significant attention in the field of materials science over the past decade due to its layered structure, resembling graphite, and exhibiting distinct anisotropic electrochemical, electronic, and optical properties. These properties make it highly relevant in various applications such as biology, physicochemistry, optics, imaging, sensing, therapy, and intercalation agents.^19–21^ Surface functionalization of MoS_2_ involves modifying its properties through the covalent bonding of particles to the single-layer nanosheets. Scanning tunneling microscopy (STM) serves as direct evidence for the covalent functionalization of transition metal dichalcogenides (TMDs), including MoS_2_.^22,23^ The functionalized MoS_2_ with a large surface area can increase its surface area and improve its effectiveness in interacting with other materials by adding functional groups or nanostructures to its surface. Its exceptional hydrophilicity provides a number of advantages, making it a promising material in a variety of biomedical applications.^24^ Overall, functionalized MoS_2_ nanostructures are important for SERS detection because they increase sensitivity, provide a large surface area for molecule adsorption, maintain chemical stability, have tunable plasmonic properties, are biocompatible, and can be easily integrated into sensing platforms.^25,26^

SnS_2_ is a layered, n-type semiconducting material with a hexagonal structure and an indirect bandgap of 2.23 eV. Similar to other TMDs, SnS_2_ consists of tin atoms sandwiched between two sulfur layers with covalent bonding, while each monolayer is held together by van der Waals forces.^27^ The structural properties of SnS_2_, including interlayer distances, binding energies, and in-plane lattice parameters, exhibit minimal variation across layers. The layer-dependent Raman spectra of SnS_2_ exhibit a slight increase in the frequencies of the Raman-active modes as the number of layers increases, while their intensities display a significant enhancement. The investigation of electronic, excitonic, and vibrational properties of SnS_2_ materials opens up new avenues for understanding the key characteristics of 2D materials.^28^ Due to its impressive performance, SnS_2_ finds applications in environmental remediation. Biofunctionalized SnS_2_ nanoparticles possess a large surface area and exceptional hydrophilicity. These attributes improve the loading efficiency and capacity of antibodies in the bio-detection stage and ensure the functionality of immobilized protein biomolecules. The functionalization of SnS_2_ is characterized using scanning electron microscopy, electrochemical impedance spectroscopy, static water contact angle measurements, and cyclic voltammetry.^29^ Because of their distinct surface properties, MoS_2_ and SnS_2_ make ideal SERS substrates. When functionalized with the MoS_2_ and SnS_2_ nanoparticles, they can greatly boost the Raman signals of molecules adsorbed on their surfaces. Charge transfer between the analytes molecule and the substrate as well as the localized surface plasmon resonance (LSPR) phenomenon are the sources of this increase. MoS_2_ and SnS_2_ nanostructures are chemically stable, which is critical for ensuring the accuracy and reliability of SERS observations throughout time. Functionalization can improve their stability and avoid degradation, resulting in more consistent and precise detection. For the sensitive and targeted identification of biomolecules, pathogens, and other analytes in complicated biological samples, functionalized MoS_2_ and SnS_2_ nanostructures can be employed.^30^

Functionalized 2D MoS_2_/SnS_2_ nanostructures for SERS have been the subject of ongoing research, particularly in nanotechnology and materials science. The functionalization of MoS_2_ economical SERS substrate for label-free bilirubin detection in clinical diagnosis. A one-pot hydrothermal synthesis of Fe-doped MoS_2_ was created as SERS substrate. Fe-MoS_2_ NFs were employed to detect bilirubin in serum. The Fe-MoS_2_ NF SERS substrate has a linear detection range of 10^−3^–10^−9^ M and a low limit of detection (LOD) of 10^−8^ M.^31^ The improvement the SERS sensitivity was also investigated of the π-conjugated fluorinated 7,7,8,8-tetracyanoquinodimethane derivatives by using the charge-localization effect caused by 2D MoS_2_ flakes. A significant Raman signal amplification in SERS was achieved using a 2D hetero structure made of F*n*TCNQ nanostructures grown on a 2D MoS_2_ flake. The SERS enhancement factor of MB molecules on the ideal F4TCNQ/MoS_2_ nanocomposite substrate can reach up to 2.531 × 10^6^, with a limit of detection (LOD) of 10^−10^ M. The SERS results for MB, Rhodamine 6G (R6G), and 4-aminothiophenol (4-ATP) molecules suggest that the F*n*TCNQ/MoS_2_ SERS platform is promising for the detection of trace molecules.^32^ The photo-assisted decorating of silver nanoparticles (Ag-NPs) by hydrothermally produced hexagonal-like tin disulfide (SnS_2_-NHs) for ultrasensitive detection of synthetic dyes. The Ag-NPs/SnS_2_ NHs nanostructure exhibits both a local electromagnetic effect from the Ag-NPs and an effective charge-transfer effect from the SnS_2_ NHs. Methylene Blue (MB) and tartrazine (TZ) were used to test the SERS performance of Ag-NPs/SnS_2_ NHs. The produced nanostructure has a large linear range (MB (10^−3^–10^−10^ M) and TZ (10^−2^–10^−9^ M), low limit of detection (MB (4.12 × 10^−10^ M) and TZ (3.01 × 10^−9^ M), and excellent enhancement factor (10^8^ and 10^7^ for MB and TZ, respectively).^33^ The simple SERS active gold functionalized SnS_2_ quantum dots (Au/SnS_2_ QDs) that can detect and photodegrade Hg^2+^ ions under visible light irradiation. Crystal violet (CV) dye is employed as the indirect Raman probe for SERS detection at an excitation laser of 532 nm. The Au hybrids had higher SERS activity than pristine-SnS_2_ due to a combination of electromagnetic and chemical enhancements. The detection limit of Au/SnS_2_ toward Hg^2+^ was determined to be 1.05 ng ml^−1^.^34,35^

In this study, we focused on investigating the surface functionalization of 2D molybdenum/tin chalcogenide nanostructures through covalent bonding. The aim was to enhance their performance, properties, and bio-compatibility. Furthermore, we explored the potential of these nanostructures as SERS substrates for the detection and identification of various analytes, including microorganisms like *Escherichia coli* (*E. coli*) Methylene Blue (MB).

## Experimental section

### Materials

The source materials used for synthesizing molybdenum disulfide/tin disulfide and for surface functionalization *via* covalent bonding in SERS applications include molybdenum trioxide (99.6%), thiourea (98.7%), hydrochloric acid (99%), tin-chlorate pentahydrate (99.2%), sodium dodecyl sulfate (99%), l-cysteine (99%), isopropyl alcohol (99%), ethanol (99.8%), and deionized water were used, as purchased from Sigma Aldrich.

### Synthesis of MoS_2_ nanoparticles

The synthesis of MoS_2_ nanoparticles was carried out using the hydrothermal method, following the procedure in the reported literature.^36^ MoO_3_ (0.15 g) and thiourea (2.0 g) were mixed by stirring for 15 minutes at 50 °C using a magnetic hot plate stirrer. Subsequently, an HCl solution (0.6 mole per l) was added to the mixture, which was then transferred to a Teflon-lined stainless steel hydrothermal autoclave. The autoclave was heated at 280 °C for 12 hours. After completion, the resulting powder was filtered, washed several times with deionized water, and dried at 100 °C. The obtained product was then annealed at 200–400 °C for 1 hour and characterized using Raman spectroscopy, X-ray diffraction (XRD), and scanning electron microscopy (SEM).

### Synthesis of SnS_2_ nanoparticles

The synthesis of SnS_2_ nanoparticles was conducted using a hydrothermal process.^37^ To begin, 1.5 g of dodecyl sulfate sodium salt was added to deionized water and stirred for 20 minutes. Subsequently, 1.7 g of SnCl_4_·5H_2_O and 1.2 g of thiourea were mixed into the aqueous solution of SDS, and the mixture was stirred for 30 minutes at mild heating. The resulting mixture was then transferred to a hydrothermal autoclave and heated at 180 °C for 12 hours. After completion, the product was filtered, washed, and dried at 100 °C, followed by annealing at 300–400 °C for 1 hour. The SnS_2_ nanoparticles were characterized using XRD, Raman spectroscopy, and SEM.

### Functionalization of MoS_2_ and SnS_2_ NPs with l-cysteine

For the functionalization of MoS_2_ and SnS_2_ with l-cysteine, the following process was conducted.^38^ Initially, we exfoliated the bulk MoS_2_ and SnS_2_ nanoparticles and took different concentrations of MoS_2_ (0.2 g/5 ml IPA, 0.8 g/5 ml IPA, 1.4 g/5 ml IPA) and SnS_2_ (0.3 g/5 ml IPA, 0.48 g/5 ml IPA, 0.58 g/5 ml IPA). These samples were sonicated under ice-cooling for one hour. Subsequently, centrifugation was performed at 4000 rpm for 1 hour, and the supernatant was discarded to remove impurities. The samples were sonicated and centrifuged again for one hour at 1500 rpm. The sediment was discarded, and the supernatant was collected for further functionalization. l-Cysteine/IPA solution was added to the centrifuged supernatants of MoS_2_ and SnS_2_ solutions, followed by sonication. The resulting dispersion was centrifuged at 9000 rpm for one hour. Equal ratios of IPA/H_2_O were added to both the MoS_2_ and SnS_2_ sediments, which were then centrifuged at 9000 rpm for 2 hours to remove free and unbound molecules from the sediment. The resulting product was filtered and dried at 100 °C. The functionalization of MoS_2_ and SnS_2_ with l-cysteine was characterized using various techniques, including Raman, XRD, SEM, and SERS.

### Preparation of MB solution

To prepare a 0.1 mM concentration of Methylene Blue, put 0.003 g of MB to a 250 ml beaker containing 100 ml DI water and stir for 10–15 minutes. Other concentrations are made using the same procedure, adding 0.32 g, 3.199 g, and 9.597 g to 100 ml DI water for 1 mM, 0.1 M, and 0.3 M, respectively.

### Preparation of *E. coli* bacteria solution

**Fig. 1:**
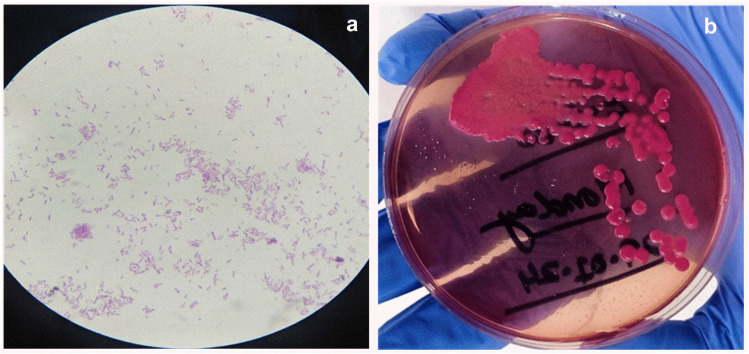
(a and b) Optical images of *E. coli* colonies.

Single bacteria colony of *E. coli* from the stock is dissolved in the 1 ml DI water. Then 0.1 ml of this solution is dissolved in 0.9 ml DI water and this processed carried for multiple time to reach the dissolution factor of 10^6^. Then 0.1 ml from each diluted solution is cultured on the Petri dish having MacConkey media and left for 24 hours. After the culturing process the dish having 61 countable colonies is carried for the calculation of CFU ml^−1^. Fig. 1(a) and b shows the optical images and real image of *E coli* bacteria colony.CFU ml^−1^ = no. of colonies × dilution factor/volume of cultured platedCFU ml^−1^ = 61 × 10^5^/0.1 mlCFU ml^−1^ = 6.1 × 10^7^ ml^−1^

This selected diluted bacteria solution is then carried for the measurements of SERS using MoS_2_-l-Cys and SnS_2_-l-Cys.

### Preparation of *E. coli* bacteria solution

*E. coli* bacteria are cultured in saline water and cultivated on MacConkey media using the steaking process. Grown colonies are collected with an inoculation loop and placed in a sterilized glass test tube containing 0.5 ml DI water. They are then shaken thoroughly to dissolve all of the bacteria in water. To make a media-free solution, it is centrifuged at least three times at 4000 rpm for 10 minutes. Gram staining is used to ensure the presence of a high concentration of bacteria in water, and optical images are acquired with an optical microscope at 100× magnification (Fig. 1(a)).

### Detection of *E. coli* bacteria by MoS_2_ and SnS_2_ as a SERS substrate

To begin with, a 100 ml solution of l-Cys-MoS_2_/l-Cys-SnS_2_ was prepared in DI water, and then 100 μl of this solution was added to 500 μl of PBS. Subsequently, 50 μl of the bacterial solution was mixed with the PBS solution. The mixture was incubated for 1 hour at 25 °C with agitation at 500 rpm, followed by centrifugation for 10 minutes at 2000 rpm. Next, the bacterial solution was subjected to three washes with PBS to eliminate any unbound particles. Finally, 2 μl of the bacterial solution was utilized for SERS observation.^39^ Comparative Raman spectrum of SERs enhancement of beard particles of MoS_2_ and SnS_2_ and functionalized particles of MoS_2_ and SnS_2_–l-Cys by using MB annealed at 400 °C, shown in Fig. S1–S3.†

## Results and discussion

The amplification of Raman signals is essential for identifying particular target compounds, including biomolecules, environmental pollutants and food chemicals.^40^ The advancement of SERS applications has largely been driven by the increasing accessibility of suitable nanostructure-based SERS substrates.^41^ In recent years, the progress in nanotechnology, electronics, lasers, and optics has led to the development of substrates with various shapes, compositions, and sizes. These substrates offer the capability to generate diverse enhancement factors (EFs) and find extensive applications in trace-level analysis. The amplification of Raman signals is influenced by both the substrate–sample interactions and the functionalization of substrates to achieve SERS-active substrates.^42^ When applying SERS in the biomolecules industry, it becomes crucial to choose appropriate functionalized substrates based on the nature and physicochemical properties of bacterial samples, considering their multi-component nature.^43^ Reproducibility, portability, sensitivity, and selectivity are among the key factors that influence SERS performance. Enhancements in these factors have played a crucial role in driving rapid progress in the development of SERS substrates. In this study we have prepared the two materials MoS_2_ and SnS_2_ which used as substrates and functionalized the substrates.

### Functionalization of MoS_2_

**Fig. 2:**
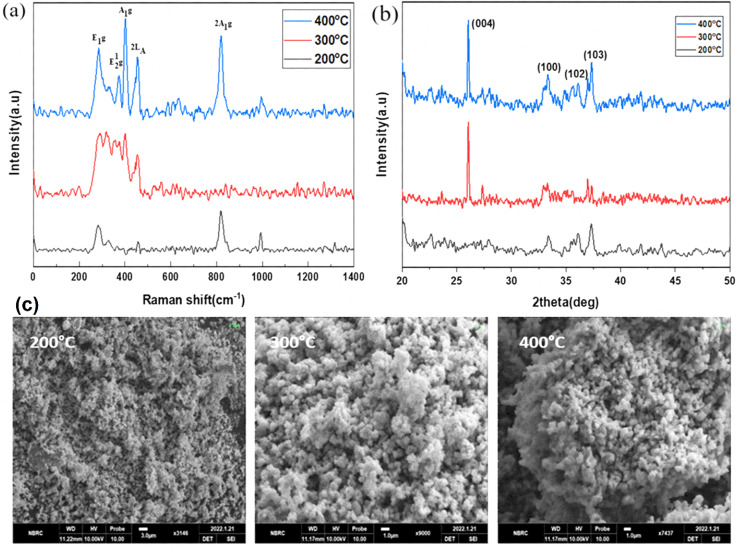
(a) Raman spectroscopy, (b) XRD, and (c) SEM analysis of annealed MoS_2_ nanoparticles at various temperatures ranging from 200 to 400 °C.

Fig. 2(a) to (c) depict the Raman, XRD, and SEM images of the MoS_2_ NPs annealed at different temperatures: 200, 300, and 400 °C, respectively. In Fig. 2(a), the Raman spectroscopy analysis explores the vibrational modes of all MoS_2_ samples and records the spectra from 200 to 1000 cm^−1^.^39^ The first peak, E_1g_, appears around 285 cm^−1^ and is Raman active in bulk 2H-MoS_2_ due to its location in the hexagonal Brillouin zone. However, since this mode is forbidden in backscattering experiments on the surface perpendicular to the *c*-axis, its presence indicates that the MoS_2_ layers are randomly oriented and not perpendicular to the laser, with no polarization. The second peak, E^1^_2g_, is observed between 373–385 cm^−1^, demonstrating a redshift phenomenon. The third peak, A_1g_, is observed at 401 cm^−1^, indicating a distinct blueshift and the presence of S-vacancies. Additionally, within the S–Mo–S layer, four active modes (E_1g_, E^1^_2g_, A_1g_) are observed as vibrational results of Raman mode shifts, and a 2LA Raman mode is also detected at 450–453 cm^−1^. We have also observed the MoO_3_ vibrational modes due to post-annealing. Because the oxygen captured ability of the molybdenum is much higher than the sulfur atoms.

In Fig. 2(b), the XRD pattern of MoS_2_ exhibits four diffraction peaks located at 2*θ* angles of 26.3°, 33.3°, 35.5°, and 38.9°, corresponding to the (004), (001), (103), and (102) planes, respectively. All of these peaks are in agreement with the JCPDS card no. 1010993. The observed broadening of the peaks confirms the pure phase and hexagonal structure of MoS_2_.

In Fig. 2(c), the SEM images of the MoS_2_ nanoparticles reveal their spherical morphology and high porosity. The agglomerations of the MoS_2_ nanoparticles were shown in the SEM images. The grain size of the nanoparticles was increased due to increase the post-annealing temperature.

**Fig. 3:**
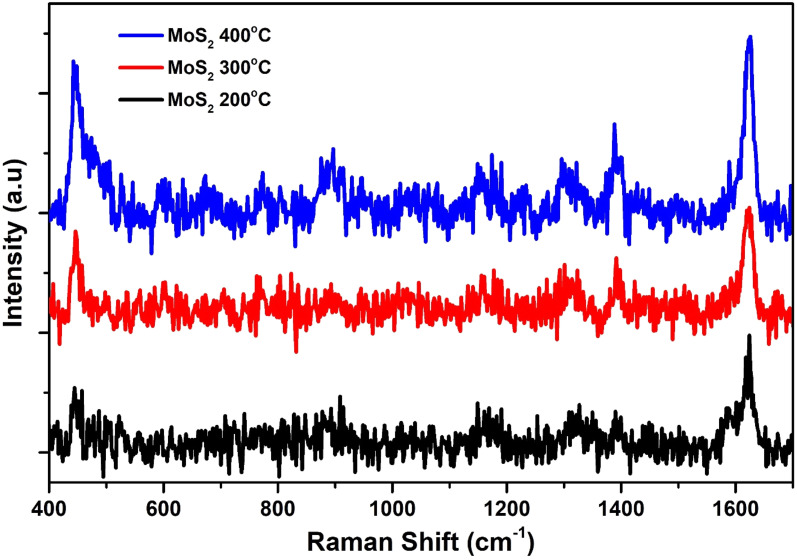
SERS spectrum of MoS_2_ annealed at temperatures ranging from 200 to 400 °C.

For the testing of synthesized nano materials SERS substrate, we have used 0.1 M of MB on the prepared sample of MoS_2_ in Fig. 3. The sample of MoS_2_ has shown high signal to noise ratio with the lower signal intensity. To overcome this issue, we functionalized our prepared samples of MoS_2_ with l-Cys.

**Fig. 4:**
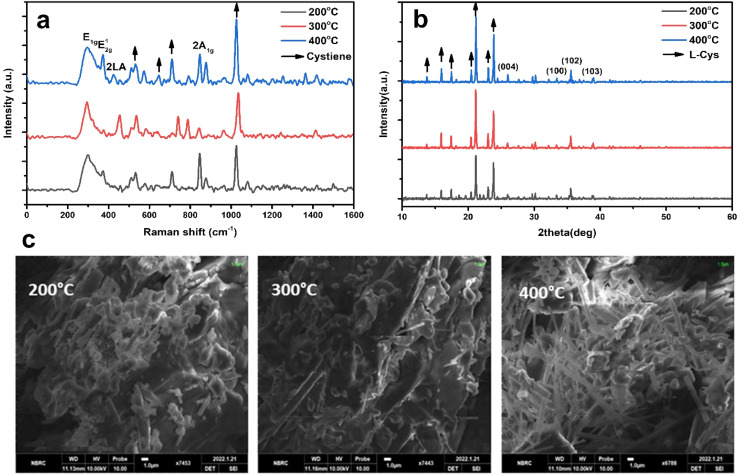
(a)–(c) Raman spectroscopy data, XRD patterns, and SEM images of the MoS_2_-l-Cys NPs annealed at temperatures ranging from 200 to 400 °C. (a) Raman spectrum (b) XRD pattern (c) SEM images.

Fig. 4(a) to (c) display the Raman, XRD, and SEM images of the MoS_2_-l-Cys NPs after functionalization, annealed at different temperatures: 200, 300, and 400 °C, respectively. In Fig. 4(a), the MoS_2_-l-Cys structure exhibits five prominent vibrational modes.^38^ Functionalization leads to an increased intensity of the E_1g_ vibrational plane, with a slight shift in position. The second vibrational mode, E^1^_2g_, remains unaffected by functionalization. Before functionalization, the results showed an intensity increase in the 2LA mode with increasing thickness. However, for MoS_2_-l-Cys NPs, the relative intensity of 2LA decreases with increasing thickness. The interaction between 2H-MoS_2_ and l-cysteine induces changes in defect density or electronic properties on the MoS_2_ surface, resulting in different band behaviors. Notably, compared to bulk 2H-MoS_2_, the MoS_2_-l-Cys structure exhibits a significant increase in the intensity of E_1g_.

In Fig. 4(b), the structural analysis of MoS_2_-l-Cys is observed using XRD. Four prominent Bragg's peaks are observed at (004), (100), (102), and (103) in addition to the peaks corresponding to l-cysteine at different angles. Upon functionalization with l-cysteine, the intensity of the MoS_2_ peaks increases; however, the sharpness of the peaks decreases.

In Fig. 4(c), the SEM analysis of MoS_2_-l-Cys NPs reveals a uniform distribution of particle sizes without any signs of aggregation.

**Fig. 5:**
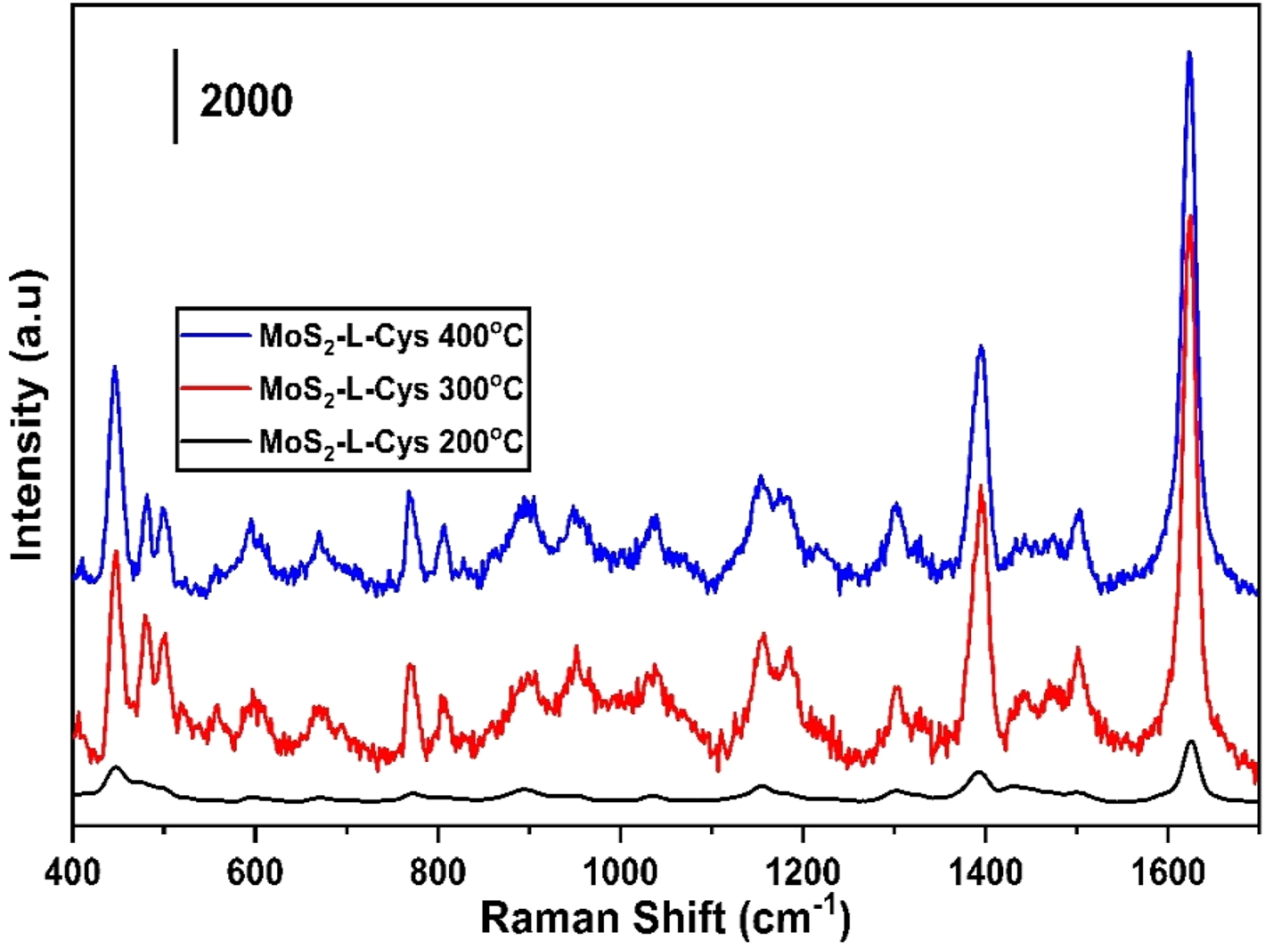
The Raman spectra of MB using MoS_2_-l-Cys as SERS substrate annealed at temperatures 200 °C, 300 °C and 400 °C.

Fig. 5 shows the SERS spectrum of 0.1 M of Methylene Blue for the testing of synthesized MoS_2_-l-Cys functionalized as a SERS substrate, which was annealed at temperatures of 200 °C, 300 °C and 400 °C. The samples annealed at 300 °C and 400 °C have shown significant enhancement in the SERS spectrum as compared to the sample annealed at 200 °C. Table 1 shows the detailed analysis of the Raman spectrum with corresponding bonds of MoS_2_.

**Table 1:** SERS spectra of MB using MoS_2_-l-Cys as the substrate for surface-enhanced Raman spectroscopy

Raman spectra of MB (cm^−1^)^this work^	Raman spectra of MB (cm^−1^)^39,43–45^	Corresponding bonds
445–479	449	C–N–C
500	502	C–N–C
671	670	C–H
769–953	768	C–H
1035	1030	C–H
1185	1184	C–N
1304	1301	C–H
1395	1396	C–H
1440	1442	C–N
1502	1513	C–C
1622	1618	C–C

**Fig. 6:**
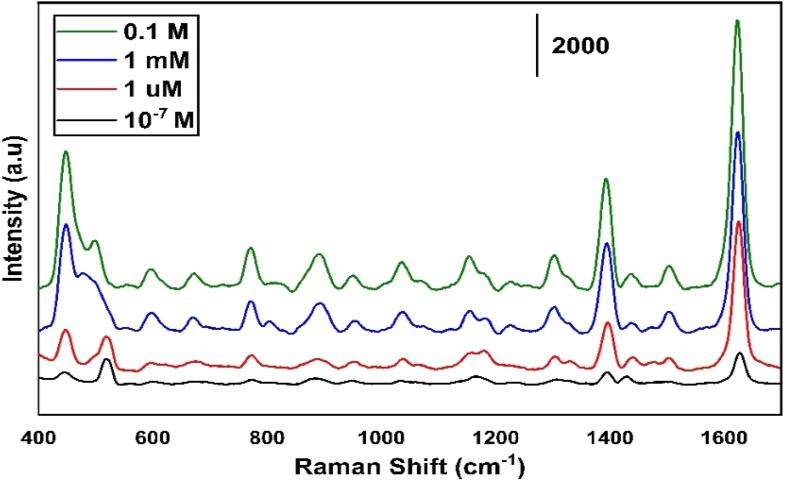
Different concentration of MB ranging from 0.1–10^−7^ M measured on MoS_2_ sample annealed at 300 °C.

Fig. 6 shows the different concentrations of MB ranging from 0.1–10^−7^ M for the least concentration of any organic molecule to be used for the SERS. Up to 1 μm concentration of MB we have observed a detectable high intensity.

**Fig. 7:**
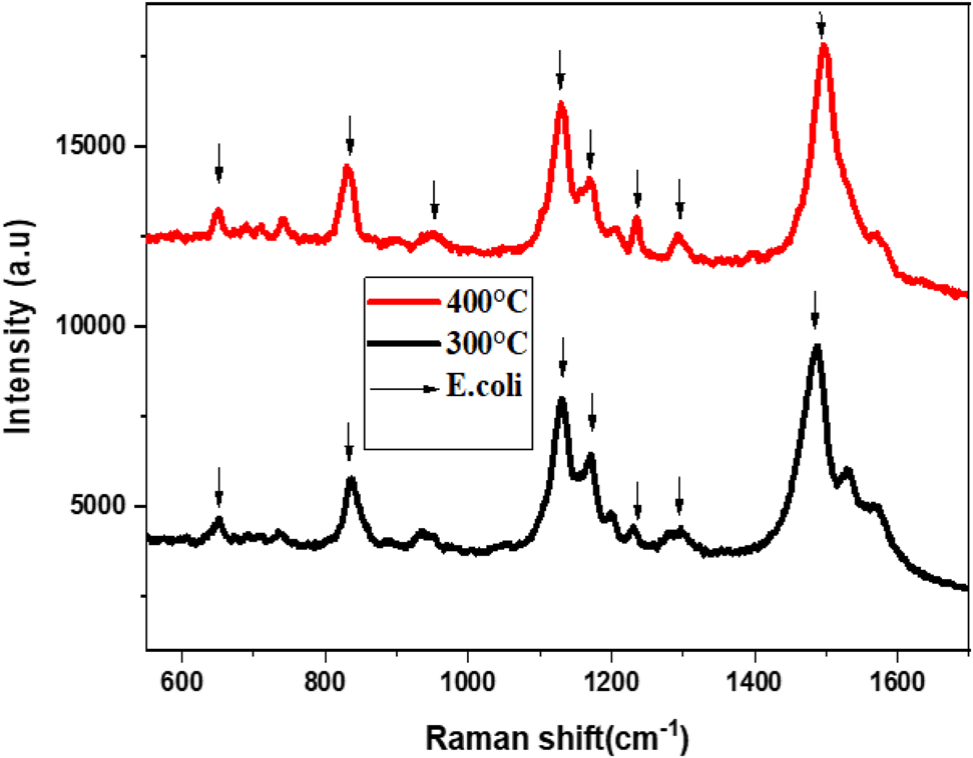
The Raman spectra of *E. coli* using MoS_2_-l-Cys as SERS substrate annealed at temperatures 300 °C and 400 °C.

Fig. 7 presents the Raman spectra of *E. coli*, which was annealed at temperatures 300 °C and 400 °C. The Raman spectra were obtained using an excitation laser of 633 nm, and eight active Raman modes were measured at wavenumbers of 652, 828, 958, 1129, 1169, 1240, 1300, 1499, and 1580 cm^−1^.

Table 2 shows the detailed analysis of the Raman spectra of *E. coli* using MoS_2_-l-Cys. The *E. coli* SERS peaks were examined for the substrates annealed at 300 °C and 400 °C. Strong peaks were observed at 828 cm^−1^ (O–P–O stretching), 1129 cm^−1^ (C=S), and 1499 cm^−1^ (carbohydrates modes). Other peaks were observed at 652 cm^−1^ (C–H), 958 cm^−1^ (CH bonding), 1169 cm^−1^ (C–C), 1240 cm^−1^ (C–N), 1300 cm^−1^ (lipids), and 1580 cm^−1^ (lipids). These results demonstrate the potential of the SERS substrate for the detection of *E. coli* bacteria even at low concentrations. However, the substrate annealed at 300 °C did not show *E. coli* signals due to a high signal-to-noise ratio. The functionalization resulted in an increase in peak intensity, indicating a change in defect density and electronic properties on the surface, as well as a slight shift in the Raman spectra. The MoS_2_-l-Cys nanoparticles were utilized as SERS substrates for the detection of *E. coli* bacterial cells, even at low concentrations. Raman analysis revealed that these substrates have the potential to detect various pathogens.

**Table 2:** SERS spectra of *E. coli* using MoS_2_-l-Cys as the substrate for surface-enhanced Raman spectroscopy

Raman spectra of *E. coli* (cm^−1^)^this work^	Raman spectra of *E. coli* (cm^−1^)^11,23,39^	Corresponding bonds
652	658	C–H
828	830	O–P–O stretch
958	960	CH bending mode
1129	1130	C=S
1169	1161	C–C
1240	1245	C–N
1300	1330	CH_2_
1499	1513	Carbohydrates
1580	1587	Lipids

### Functionalization of SnS_2_

**Fig. 8:**
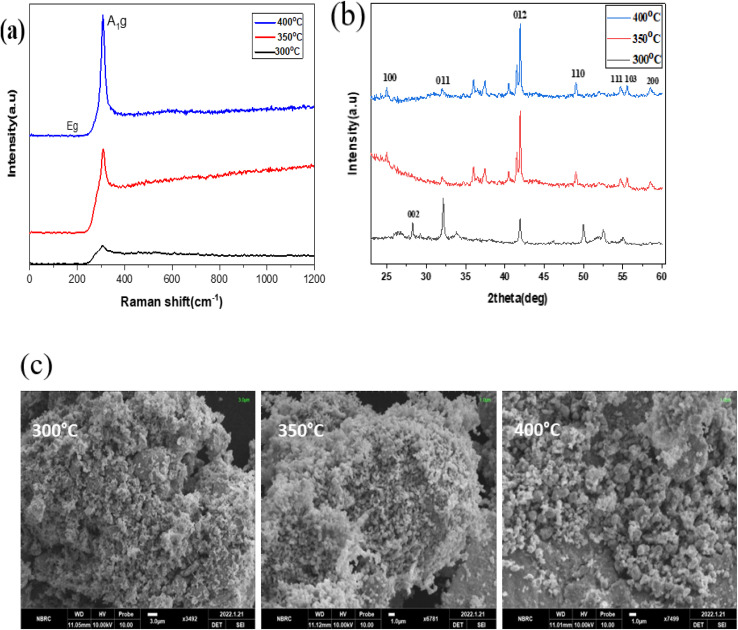
(a)–(c) XRD, Raman spectroscopy data, and SEM images of the SnS_2_ NPs annealed at different temperatures ranging from 300 to 400 °C.

Fig. 8(a) to (c) depict the Raman, XRD, and SEM images of the SnS_2_ NPs annealed at different temperatures: 300, 350, and 400 °C, respectively. In Fig. 8(a), the Raman analysis focuses on the first peak at 202 cm^−1^, which corresponds to SnS, and the main peak at 312 cm^−1^ for SnS_2_.^44^ The Raman spectra of SnS_2_ exhibit a pronounced peak at 312 cm^−1^ in the A_1g_ mode, indicating the stretching of sulfur atoms out of the plane. The in-plane stretched mode at 204 cm^−1^ (E_g_) appears weak due to the reduced number of in-plane scattering events caused by the nano size effect. The Raman spectra of SnS_2_ display multiple peaks, but the absence of a peak at 633 cm^−1^ indicates that SnS_2_ has not been oxidized to SnO_2_. Furthermore, two peaks corresponding to A_1u_ and A_1g_-LA were observed, suggesting the presence of thick layers of SnS_2_ in the samples.

In Fig. 8(b), the purity and phase of the SnS_2_ samples are determined through the XRD pattern. The XRD pattern of SnS_2_ at different temperature ranges reveals distinct diffraction peaks. At 300 °C, the peak is consistently indexed as a hexagonal SnS_2_ phase. As the temperature decreases, the formation of both SnS and SnS_2_ is observed. The XRD analysis of SnS_2_ shows seven reflection peaks corresponding to (100), (002), (011), (012), (110), (111), (103), and (200) planes.

In Fig. 8(c), the morphology of the prepared SnS_2_ samples was examined using SEM analysis. The SEM investigation revealed that SnS_2_ exhibits nanoflakes with a hexagonal stacking structure.

**Fig. 9:**
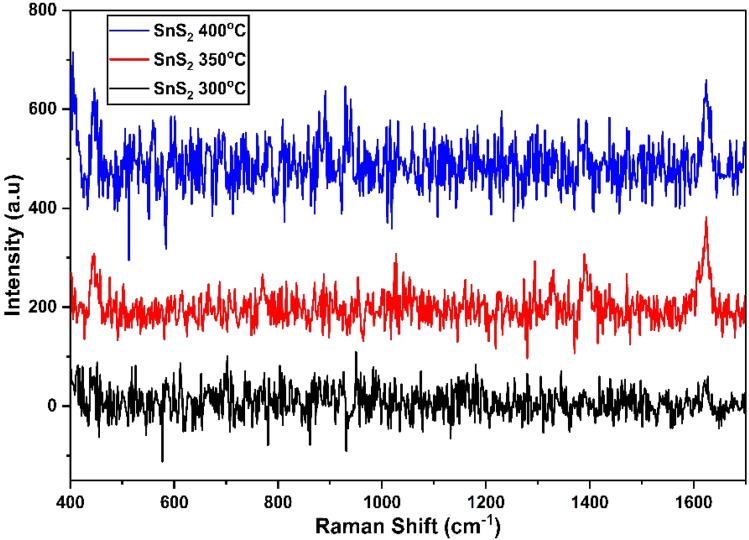
SERS spectrum of SnS_2_ annealed at temperatures ranging from 300 to 400 °C.

We have used 0.1 M of MB on the prepared sample of MoS_2_ in Fig. 9 for the testing of synthesized nano materials SERS substrate. Fig. 9 shows that the sample of SnS_2_ has shown a higher signal-to-noise ratio with a lower signal intensity. To overcome this issue we functionalized our prepared samples of SnS_2_ with l-Cys.

**Fig. 10:**
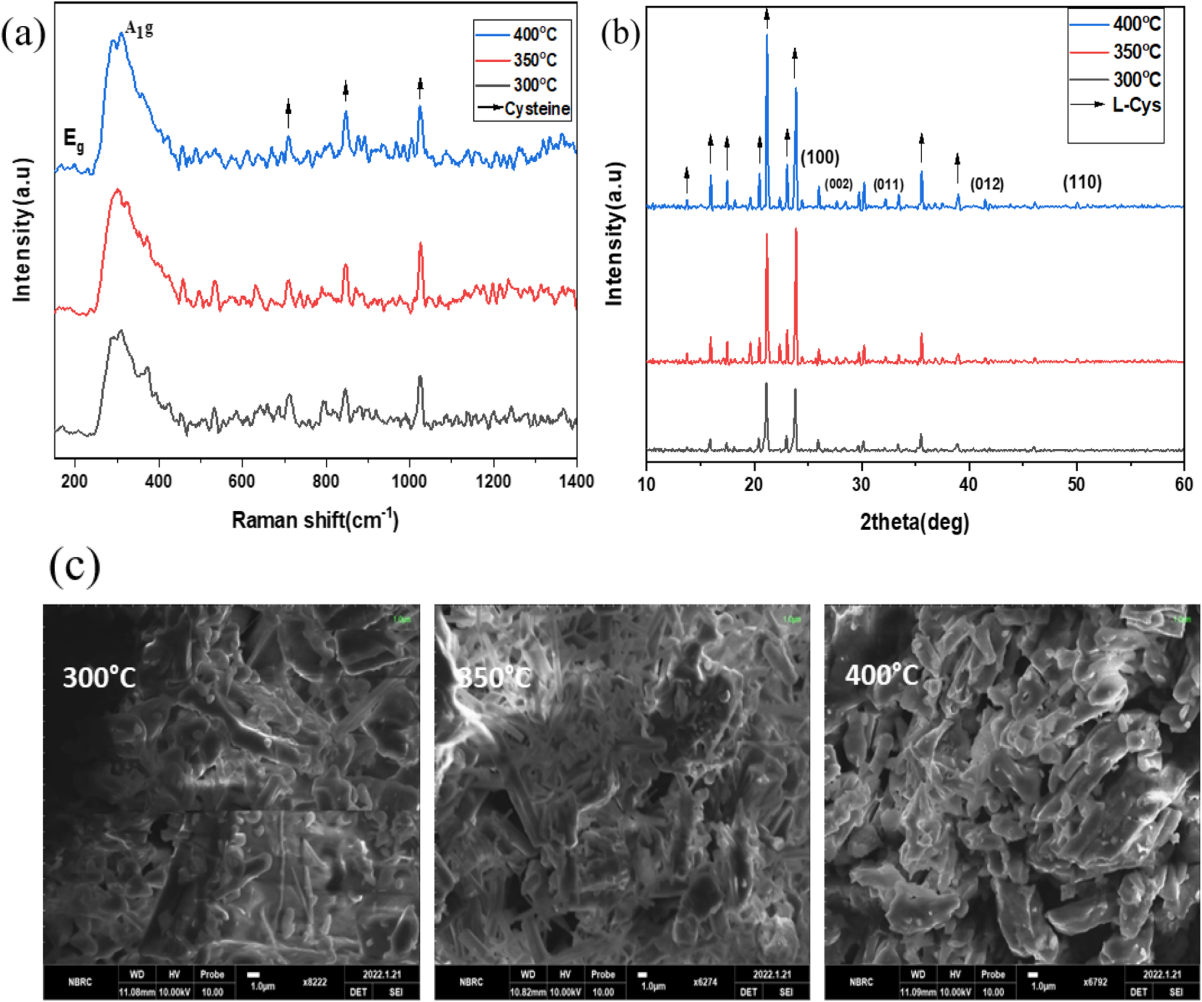
(a)–(c) XRD, Raman spectroscopy data, and SEM images of the SnS_2_-l-Cys NPs annealed at different temperatures ranging from 300 to 400 °C.

Fig. 10(a) to (c) depict the Raman, XRD, and SEM images of the SnS_2_-l-Cys NPs annealed at different temperatures ranging from 300 to 400 °C. In Fig. 10(a), the Raman spectra of SnS_2_ exhibit a slight shift in their peaks. The interaction between SnS_2_ and l-cysteine is evident from the presence of the E_g_ peak at 204 cm^−1^, which is still weak due to the nano size effect. The Raman spectra at 312 cm^−1^ indicate the presence of the stretched out-plane peak mode, but a shift in position is observed after functionalization. l-Cysteine peaks at 720 cm^−1^, 843 cm^−1^, and 1020 cm^−1^ were observed at different temperatures, respectively. SnS_2_ with l-cysteine displayed Raman spectra that were not significantly different from the SnS_2_ spectra, but the interaction with l-cysteine affected the peak positions.

In Fig. 10(b), after functionalization of SnS_2_ with l-cysteine, five diffraction peaks corresponding to (100), (002), (011), (012), and (110) were observed, along with l-cysteine peaks at different angles and with varying intensities. The X-ray diffraction (XRD) pattern was used to determine the purity and phase of SnS_2_-l-cysteine. The intensity of the peaks increased after functionalization.

The SEM micro-images in Fig. 10(c) display the SnS_2_-l-Cys NPs annealed at different temperatures ranging from 300 to 400 °C. Following functionalization, the original morphology of both SnS_2_ and l-Cys elements is no longer apparent, and instead, a uniform distribution of particle sizes is observed.

**Fig. 11:**
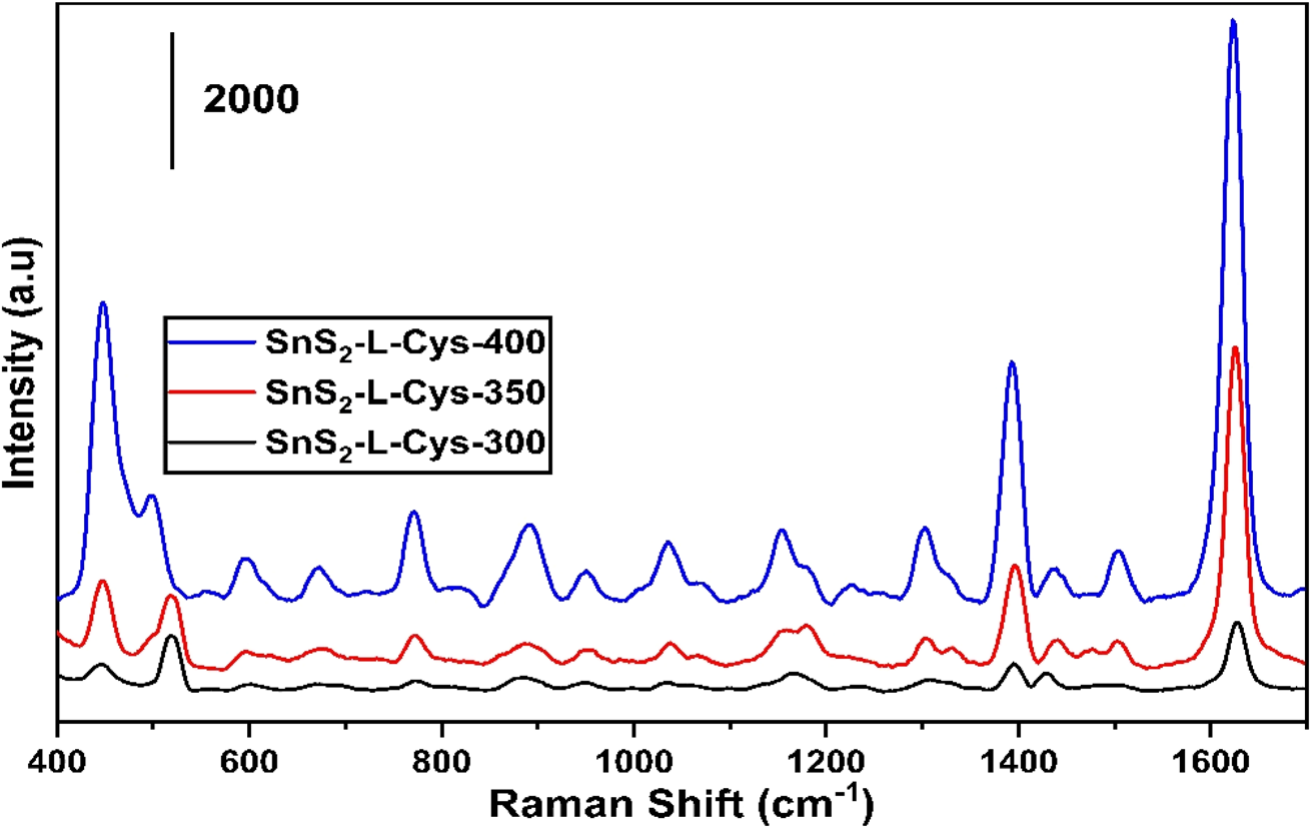
SERS spectrum of SnS_2_-l-Cys annealed at different temperatures ranging from 300 to 400 °C.

Fig. 11 illustrates the SERS spectrum of 0.1 M of Methylene Blue for the testing of synthesized SnS_2_-l-Cys functionalized as a SERS substrate, which was annealed at the temperatures of 300 °C, 350 °C and 400 °C. The samples annealed at 350 °C and 400 °C have shown significant enhancement in the SERS spectrum as compared to the sample annealed at 300 °C. Table 3 shows the Raman analysis of MB using SnS_2_-l-Cys.

**Table 3:** SERS spectra of MB using SnS_2_-l-Cys as the substrate for surface-enhanced Raman spectroscopy

Raman spectra of MB (cm^−1^)^this work^	Raman spectra of MB (cm^−1^)^39,43–45^	Corresponding bonds
448	449	C–N–C
500	502	C–N–C
675	670	C–H
773–949	768	C–H
1036	1030	C–H
1185	1184	C–N
1302	1301	C–H
1392	1396	C–H
1440	1442	C–N
1505	1513	C–C
1626	1618	C–C

**Fig. 12:**
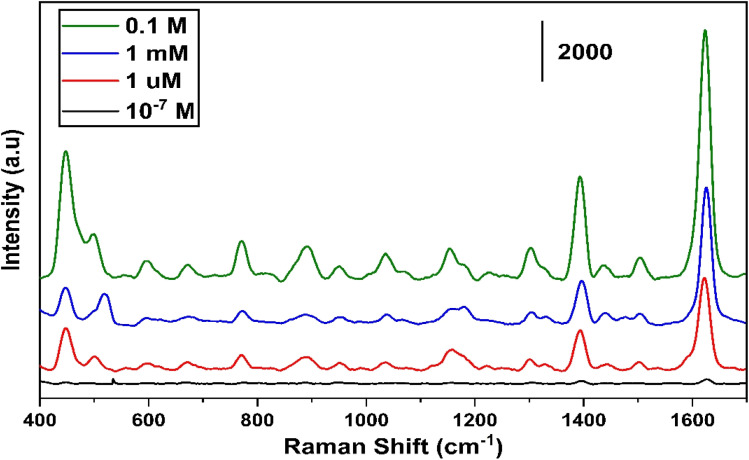
Different concentrations of MB ranging from 0.1–10^−7^ M measured on SnS_2_ sample annealed at 400 °C.

Fig. 12 illustrates the tested results for different concentrations of MB ranging from 0.1–10^−7^ M for the least concentration of any organic molecule to be used for the SERS. The maximum 1 μm concentration of MB we have observed a detectable enhancement in the intensity of Raman signal.

**Fig. 13:**
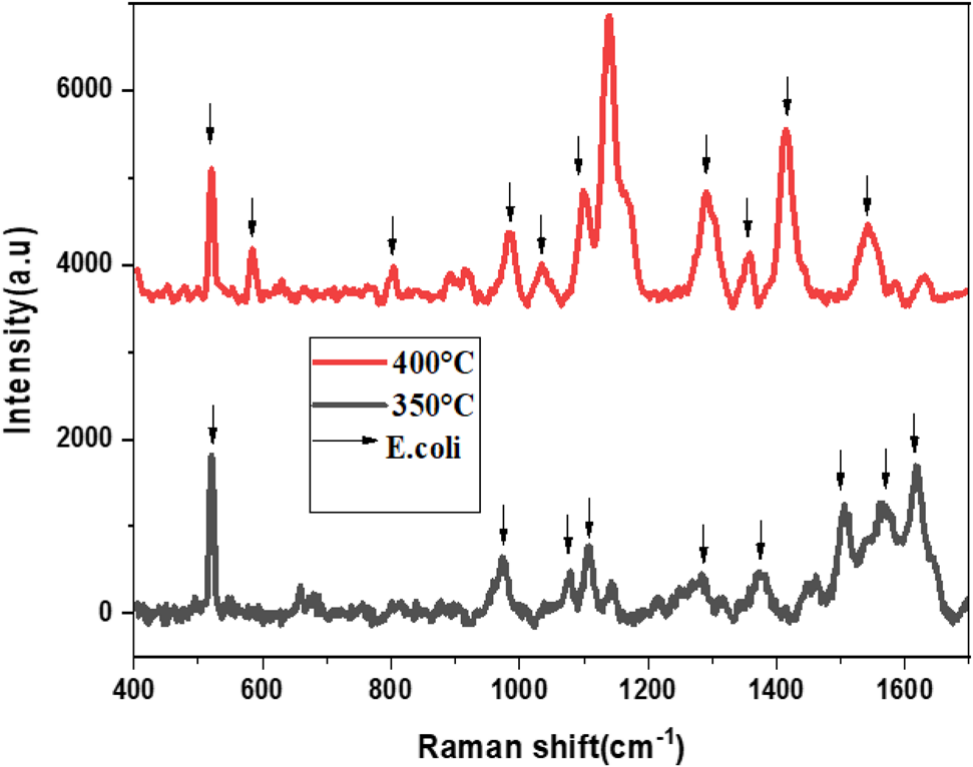
The Raman spectra of *E. coli* using SnS_2_-l-Cys as SERS substrate annealed at different temperatures ranging from 300 to 400 °C.

In Fig. 13, the Raman spectra of *E. coli* using SnS_2_–l-Cys as a SERS substrate annealed at high temperatures, specifically 350 °C and 400 °C, are presented. Eleven active Raman modes were identified by utilizing a 633 nm excitation laser. These modes correspond to vibrations at 520, 582, 803, 983, 1034, 1098, 1137, 1290, 1340, 1400, and 1540 cm^−1^, which are associated with C–N–C, S–S, tyrosine, C–O–O, C–H, C=S, CH_2_, NH_2_, and ring stretching of adenine, respectively. The results demonstrate that this substrate can be used for the detection of *E. coli* bacteria at low concentrations.

The functionalization increased in peak intensity, indicating a change in defect density and electronic properties on the surface, as well as a slight shift in the Raman spectra. The annealing temperature has a considerable impact on the characteristics of functionalized SnS_2_-l-cysteine SERS substrates. Higher annealing temperatures can improve the homogeneity and reproducibility of SERS signals and enhancement factors can also be affected by its annealing temperature which are critical for detecting low-concentration analytes in SERS applications. The adsorption and binding of molecules, such as the l-cysteine–SnS_2_ substrate surface are influenced by the annealing temperature. The higher temperatures of 400 °C can enable greater adsorption or binding interactions, improving the functionalized substrate stability, and increasing the intensity of the SERS signal.

The SnS_2_-l-Cys nanoparticles were utilized as SERS substrates for the detection of *E. coli* bacterial cells, even at low concentrations. Raman analysis revealed that these substrates have the potential to detect various pathogens. Table 4 shows the detailed analysis of Raman spectra of *E. coli* using SnS_2_–l-Cys as an SERS substrate.

**Table 4:** SERS spectra of *E. coli* using SnS_2_–l-Cys as SERS substrate

Raman spectra of *E. coli* (cm^−1^)^this work^	Raman spectra of *E. coli* (cm^−1^)^39,43,44^	Corresponding bonds
520	525	C–N–C
582	580	S–S
803	796–809	Tyrosine
983	960–996	C–O–O
1034	1030	C–H
1098	1100	Aromatic C=S
1137	1126–1144	C=S
1290	1300	CH_2_
1340	1336–1342	γ(NH_2_)
1400	1396	C–H
1540	1551–1569	Ring stretching (adenine)

## Conclusion

In conclusion, this study successfully functionalized 2D MoS_2_ and SnS_2_ nanostructures with l-cysteine, enhancing their performance, bio-compatibility, and structural properties. Characterization using Raman, XRD, SEM, and SERS confirmed effective covalent bonding and improved morphology. The functionalized nanostructures proved to be effective SERS substrates for detecting *Escherichia coli*, demonstrating their potential for applications in food safety, medical diagnostics, and environmental monitoring. Future research could optimize the functionalization process for improved sensitivity and explore their use with other analytes, further expanding their practical applications.

## Data availability

All data is available on the request.

## Author contributions

Zainab Ishfaq: writing original draft, Layla A. Almutairi: formal analysis, funding, M. Yasir Ali: conceptualization, Salhah Hamed Alrefaee: formal analysis, Mohamed Abdelsabour Fahmy: resources, Abdul Mateen: experiment section, Elsammani Ali Shokralla: data collection, Lamiaa G. Alharbe: review and editing, Adnan Ali: supervisor, conceptualization, Arslan Ashfaq: review & editing, conceptualization, A. R. Abd-Elwahed: frmal analysis.

## Conflicts of interest

There are no conflicts of interest.

## Supplementary Material

RA-014-D4RA05315J-s001
