# Supplementary material for: Enhanced surface functionalization of 2D molybdenum/tin chalcogenide nanostructures for effective SERS detection of Escherichia coli
Source: RSC Adv. 2024 Nov 4;14(47):35021–34. doi: 10.1039/d4ra05315j (PMC11533536; doi:10.1039/d4ra05315j)
Supplement: RA-014-D4RA05315J-s001 [file RA-014-D4RA05315J-s001.pdf]

Supplementary Information (SI) for RSC Advances.
This journal is © The Royal Society of Chemistry 2024

Enhanced Surface Functionalization of 2D Molybdenum/Tin Chalcogenide Nanostructures for Effective SERS Detection of *Escherichia coli*

Zainab Ishfaq[a], Layla A. Almutairi[b], M. Yasir Ali[a], Salhah Hamed Alrefaee[c], Mohamed Abdelsabour Fahmy[d, e], Elsammani Ali Shokralla[f], Lamiaa G. Alharbe[g], Adnan Ali[a], Arslan Ashfaq[a*], A.R. Abd-Elwahed[h,i]*

[a]*Department of Physics, Government College University, Faisalabad, 38000, Pakistan*
[b]*Department of Biology, College of Science Princess Nourah bint Abdulrahman University, P.O. Box 84428, Riyadh 11671, Saudi Arabia*
[c]*Department of Chemistry, Faculty of Science, Taibah University, Yanbu 30799, Saudi Arabia*
[d]*Adham University College, Umm Al-Qura University, Adham 28653, Makkah, Saudi Arabia*
[e]*Faculty of Computers and Informatics, Suez Canal University, New Campus, 41522 Ismailia, Egypt*
[f]*Department of Physics, Faculty of Science, Al-Baha University, Alaqiq, 65779-7738, Saudi Arabia*
[g]*Department of Physics, Aljamoum University College, Umm Al-Qura University, Makkah, Saudi Arabia*
[h]*Department of Physics, College of Science, Qassim University, Buraydah 51452, Saudi Arabia*

Corresponding Authors[*]: *arslan.ashfaq201@gmail.com (Arslan Ashfaq)*

adnnan_1982@yahoo.com (Adnan Ali)

Supplementary data

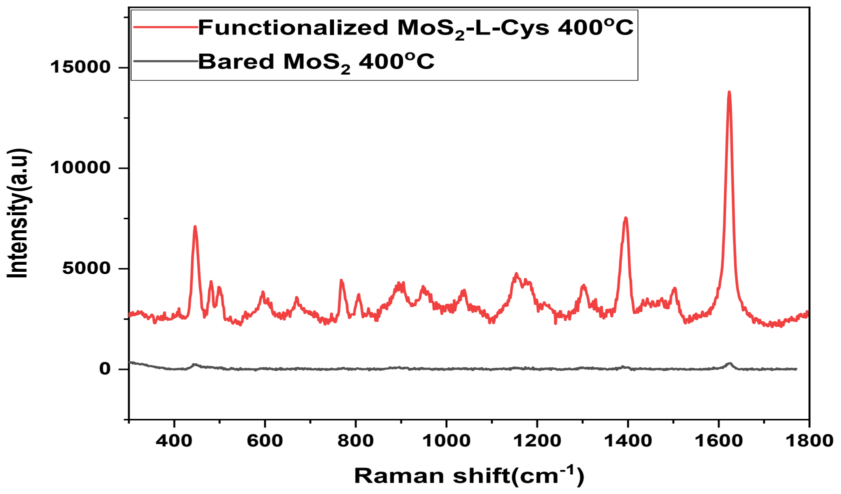

Figure S1. The Comparative Raman spectrum of SERs enhancement of beard particles of MoS_2 and functionalized particles of MoS_2-L-Cys by using MB annealed at 400°C.

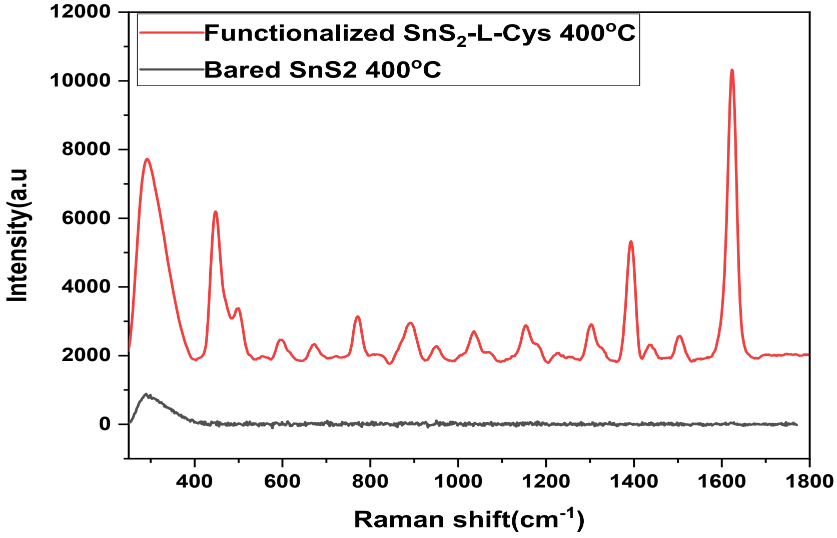

Figure S2. The Comparative Raman spectrum of SERs enhancement of beard particles of SnS_2 and functionalized particles of SnS_2-L-Cys by using MB annealed at 400°C.

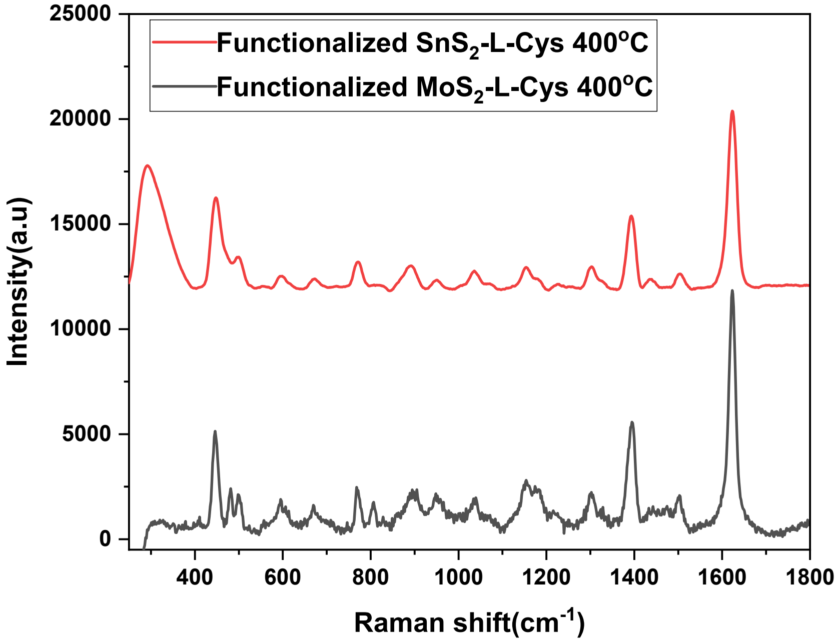

Figure S3. The Comparative Raman spectrum of SERs enhancement of functionalized particles of MoS2-L-Cys and SnS_2-L-Cys by using MB annealed at 400°C.
